# Interclonal Variations in the Molecular Karyotype of *Trypanosoma cruzi*: Chromosome Rearrangements in a Single Cell-Derived Clone of the G Strain

**DOI:** 10.1371/journal.pone.0063738

**Published:** 2013-05-07

**Authors:** Fabio Mitsuo Lima, Renata Torres Souza, Fábio Rinaldo Santori, Michele Fernandes Santos, Danielle Rodrigues Cortez, Roberto Moraes Barros, Maria Isabel Cano, Helder Magno Silva Valadares, Andréa Mara Macedo, Renato Arruda Mortara, José Franco da Silveira

**Affiliations:** 1 Departamento de Microbiologia, Imunologia e Parasitologia, Escola Paulista de Medicina, Universidade Federal de São Paulo, São Paulo, São Paulo, Brazil; 2 Skirball Institute of Biomolecular Medicine, New York University Cancer Center, New York University School of Medicine, New York, New York, United States of America; 3 Departamento de Genética, Instituto de Biociências, Universidade Estadual Paulista Júlio de Mesquita Filho, Botucatu, São Paulo, Brazil; 4 Departamento de Bioquímica e Imunologia, Instituto de Ciências Biológicas, Universidade Federal de Minas Gerais, Belo Horizonte, Minas Gerais, Brazil; 5 Campus Centro-Oeste Dona Lindu, Universidade Federal de São João del-Rey, Divinópolis, Minas Gerais, Brazil; Instituto Butantan, Laboratório Especial de Toxinologia Aplicada, Brazil

## Abstract

*Trypanosoma cruzi* comprises a pool of populations which are genetically diverse in terms of DNA content, growth and infectivity. Inter- and intra-strain karyotype heterogeneities have been reported, suggesting that chromosomal rearrangements occurred during the evolution of this parasite. Clone D11 is a single-cell-derived clone of the *T. cruzi* G strain selected by the minimal dilution method and by infecting Vero cells with metacyclic trypomastigotes. Here we report that the karyotype of clone D11 differs from that of the G strain in both number and size of chromosomal bands. Large chromosomal rearrangement was observed in the chromosomes carrying the tubulin loci. However, most of the chromosome length polymorphisms were of small amplitude, and the absence of one band in clone D11 in relation to its reference position in the G strain could be correlated to the presence of a novel band migrating above or below this position. Despite the presence of chromosomal polymorphism, large syntenic groups were conserved between the isolates. The appearance of new chromosomal bands in clone D11 could be explained by chromosome fusion followed by a chromosome break or interchromosomal exchange of large DNA segments. Our results also suggest that telomeric regions are involved in this process. The variant represented by clone D11 could have been induced by the stress of the cloning procedure or could, as has been suggested for *Leishmania infantum,* have emerged from a multiclonal, mosaic parasite population submitted to frequent DNA amplification/deletion events, leading to a 'mosaic' structure with different individuals having differently sized versions of the same chromosomes. If this is the case, the variant represented by clone D11 would be better adapted to survive the stress induced by cloning, which includes intracellular development in the mammalian cell. Karyotype polymorphism could be part of the *T. cruzi* arsenal for responding to environmental pressure.

## Introduction

The flagellate protozoan *Trypanosoma cruzi*, the etiologic agent of Chagas' disease, comprises a pool of populations which circulate in domestic and sylvatic cycles involving humans, insect vectors and animal reservoirs [1], [2]. Natural populations of *T. cruzi* are genetically diverse in terms of DNA content, isoenzyme profiles, size, growth and infectivity [1]. The absence in *T. cruzi* of detectable sexual reproduction and chromosome condensation during the cell cycle precludes classical cytogenetics analysis of the parasite. Using pulsed field gel electrophoresis (PFGE) it has been demonstrated that the parasite exhibits extensive chromosomal polymorphism [3]–[9]. Inter- and intra-strain karyotype heterogeneities suggest that chromosomal rearrangements occurred during the evolution of this parasite [5], [6], [8], [9]. The first evidence of intra-strain chromosomal heterogeneity was reported by McDaniel and Dvorak (1993) in naturally occurring variants of the Y-02 stock of the *T. cruzi* Y strain [10]. They found chromosome and gene rearrangements among Y strain stocks, confirming the extensive plasticity of the *T. cruzi* genome.

D11 is a single-cell-derived clone of the G strain of *T. cruzi* obtained in our laboratory by the limiting dilution method [11]. Vero cells were infected with metacyclic trypomastigotes of the G strain, and the selected clones were expanded by infecting naive Vero cells. Cell invasion assays using extracellular amastigote forms [12], [13] showed that clone D11 was approximately 10–15% less infective for HeLa cells than its parental G strain [14]. Taken together, these data suggest the existence of phenotypic and genotypic differences in biological properties between clone D11 and the parental G strain.

Preliminary results based on karyotypic analysis have already shown that clone D11 differs from the parental G strain in both the number and size of chromosomes. Here we show that these differences are probably due to chromosomal rearrangements. We attempt to elucidate whether these chromosomal rearrangements occurred during the cloning process and/or if they were the result of the selection of a subpopulation from the original uncloned strain. For this, we also address other questions: 1) what is the contribution of genome size and repetitive DNA content to the chromosomal polymorphism observed in clone D11? and 2) what is the synteny level between clone D11 and the G strain when large homologous chromosomal segments are examined? The results described in this paper demonstrate the existence of chromosomal rearrangements in single-cell-derived clones of the G strain of *T. cruzi*.

## Materials and Methods

### Parasites

The G strain (*Trypanosoma cruzi* group I - TcI) was isolated by Mena Barreto from an opossum in the Brazilian Amazon. It was originally introduced in our laboratory in the early 1980s by Nobuko Yoshida (obtained from Erney P. Camargo), who described the corresponding metacyclic trypomastigote forms [15]. Parasites were maintained by alternate cyclic passages in mice and LIT medium. After seven days, an aliquot of the culture was transferred to a fresh medium in a ratio of 1 10. Metacyclic trypomastigotes were harvested from cultures in the stationary growth phase and purified by chromatography on a DEAE-cellulose column, as previously described [15].

The G strain was cloned [11] following the procedure described by [16]. Vero cells grown in 96 wells plates were infected with 0.5 parasites/well (metacyclic trypomastigotes of the original G strain). The plates were then monitored for the appearance of tissue culture trypomastigotes in the supernatants. After 14 to 30 days, the parasites derived from single wells that did not have a positive neighbor well were assumed to be clones and subsequently used to re-infect Vero cells for expansion and re-cloning. After this procedure, they were frozen as epimastigotes. In this cloning procedure, 6 clones were obtained from four 96-well plates. Preliminary assessment of their biological properties (infectivity *in vitro* and *in vivo*, *in vitro* growth curves, metacyclogenesis, expression of the parental G strain major glycoproteins gp90, gp82 and 35/50 mucins) showed that only clone D11 differed from the parental strain. Therefore, it was selected for further molecular characterization and then subsequentially maintained exactly as the parental G strain (LIT/mice).

### Genetic profiling based on amplifications of the 24Sα rDNA gene and sequences from microsatellite loci

Microsatellite analysis was performed using sequences from 10 microsatellite loci previously described [17], [18]. Five consist of dinucleotide repeats (MCLE01, MCLF10, MCLG10, SCLE10 and SCLE11), four are composed of trinucleotide repeats (TcATT14, TcTAT20, TcTAC15 and TcAAT8) and one is composed of tetranucleotide repeats (TcAAAT6). The PCR assays were performed as described previously by [18]. To determine clone D11 allele sizes, 1 to 3 µL of each of the PCR fluorescent products were run on a 6% denaturing polyacrylamide gel and analyzed with an ALF DNA sequencer (GE Healthcare, Milwaukee, WI), and the fragments were compared with fluorescent DNA fragments of 50 bp to 500 bp by using the Allele Locator software (GE Healthcare) to determine their sizes.

Amplification of the D7 domain of the 24Sα rDNA gene was achieved by PCR with primers D71 (5′-AAGGTGCGTCGACAGTGTGG-3′) and D72 (5′-TTTTCAGAATGGCCGAACAGT-3′) by following the protocols described previously by [19]. Next, 5 µL of the PCR products were run on a 6% polyacrylamide gel and silver stained. DNA of *T. cruzi* strains and clones belonging to *T. cruzi* I (Col1.7G2, rDNA type 2–110 bp), *T. cruzi* II (JG, rDNA type 1–125 bp) and *T. cruzi* V (SO3 cl5, rDNA type 1/2- 110/125 bp) were used as references for 24Sα rDNA profiles.

### Measurement of genome sizes

The genome sizes of clone D11 and the G strain were determined as described previously [9]. Briefly, epimastigotes from these two isolates were synchronized with 20 mM hydroxyurea. Then, total DNA from each isolate was extracted from 10^8^ cells and subsequently quantified using a fluorescent double stranded DNA stain. Five independent experiments were performed. The ANOVA test was performed with GraphPad InStat version 3.05 software, and statistical significance was set at P<0.05.

### Estimation of repetitive sequence copy number

Genomic DNA from G strain and clone D11 was denatured with 0.4 M NaOH for 10 minutes, chilled on ice and diluted with an equal volume of 2 M ammonium acetate. Increasing amounts of DNA (62.5 ng, 125 ng, 250 ng, 500 ng e 1000 ng) were loaded onto nylon membrane (GE Healthcare) using a dot-blot apparatus (Bio-Rad) coupled with a vacuum pump and exposed to ultraviolet radiation in a UVC 500 Crosslinker (Amersham).

Recombinant plasmids containing repeated sequences were loaded on the same membranes to construct a standard curve. For this, the following recombinant plasmids were used: F4.10, which carries 3.3 units of satellite DNA (GenBank accession number AY520076); F3.17 which carries part of non-LTR retrotransposon L1Tc (GenBank accession number X83098); C6 interspersed DNA element (GenBank accession number U16295) similar to SIRE; and TcTREZO, a site-specific repeated element (GenBank accession number AF508945). pUC18 was used as a background control. Filters were hybridized in exactly the same way as the chromoblots. After hybridization procedure the amount of ^32^P in each spot was determined by liquid scintillation counting and compared with ^32^P values obtained from standards. The copy number of each repeated sequence was calculated based on genome size of each isolate.

### Pulsed-field gel electrophoresis (PFGE) and hybridization

Log-phase epimastigotes were washed in phosphate buffered saline and collected by centrifugation, and an equal volume of cell suspension was mixed with 2% low-melting temperature agarose as previously described [9].

Chromosomal DNA was separated by PFGE and hybridized with the probes indicated in the text, as described [9], [20].

### Restriction enzyme analysis

For α-tubulin gene loci and telomere length analysis, 5 µg of total genomic DNA were digested with *Pac*I and *Sfi*I and *Hae*III and *Msp*I restriction enzymes (10 U), respectively. After incubation for 2 h at 37°C, restriction fragments were submitted to unidirectional electrophoresis on a 0.8% agarose gel followed by staining with 0.5 mg/mL EtBr. The fractionated DNAs were then transferred to nylon membranes and hybridized with the selected probes.

For megarestriction analysis of α-tubulin gene loci, plugs containing chromosomes were separated by PFGE as described above, and the 5 mm agarose blocks containing the resolved chromosomal bands were then excised from the gel. Blocks were washed in TE buffer (10 mM Tris-HCl, pH 8, 1 mM EDTA) at 4°C and then equilibrated in restriction enzyme buffer before being incubated with 3,000 U of *Pac*I and *Sfi*I at 37°C for 6 hours. The restriction fragments were fractionated in a contour-clamped homogeneous electric field apparatus (Bio-Rad, CA) in a 1.1% agarose-0.5X TBE gel for 18 hours at 14°C and submitted to a voltage of 6 V/cm and a linear gradient of switching times from 30 to 70 s. Fragments were then transferred to a nylon membrane and hybridized with an α-tubulin radiolabeled probe, as described above.

## Results

### Genome size variations between the parental G strain and clone D11

Epimastigotes of the G strain and clone D11 were arrested with hydroxyurea in the G1/S- cell cycle phase (Figure S1) and the total DNA content per cell (nucleus and kDNA) estimated by means of the dsDNA quantitation method [9]. The mean total DNA (nucleus and kDNA) contents of parasites from the G strain and G-strain derived clone D11 were, respectively, 0.122270±0.026692 and 0.110409±0.015009 pg per cell. Variance analysis (ANOVA test) was performed to detect significant differences between the isolates. Although the G strain genome is slightly larger than the clone D11 genome (around 10 Mb), the difference was not statistically significant (P>0.05). The nuclear genome size was determined for each isolate based on the assumption that kDNA accounts for 20–25% of the parasite's total DNA [21]. The sizes of the clone D11 and the G strain nuclear genomes were estimated to be 81.0 and 89.8 Mb, respectively.

The amount of repetitive DNA sequences may be an important factor in determining variation in genome sizes. Therefore, we compared the G strain and clone D11 in relation to the copy numbers of four *T. cruzi* species-specific repetitive DNA sequences: a highly repetitive sequence (195 bp satellite DNA element), three middle-copy number sequences, two non-LTR retrotransposons (L1Tc and C6) and a site specific repetitive element (TcTREZO) [22]. The copy number of satellite DNA per cell was estimated at 9,341 sequences, or 2.0% of the genome of the G strain, and 10,673 sequences, or 2.6% of the genome of clone D11. The copy number estimated for TcTREZO was 2,896 and 1,855 sequences per cell in clone D11 and the G strain, respectively. The corresponding figures for the retrotransposons L1Tc and C6 were estimated to be 323 and 400 copies in the G strain and 423 and 541 copies in clone D11, respectively.

### Molecular karyotype and characterization of rearranged chromosomes by hybridization

**Figure 1:**
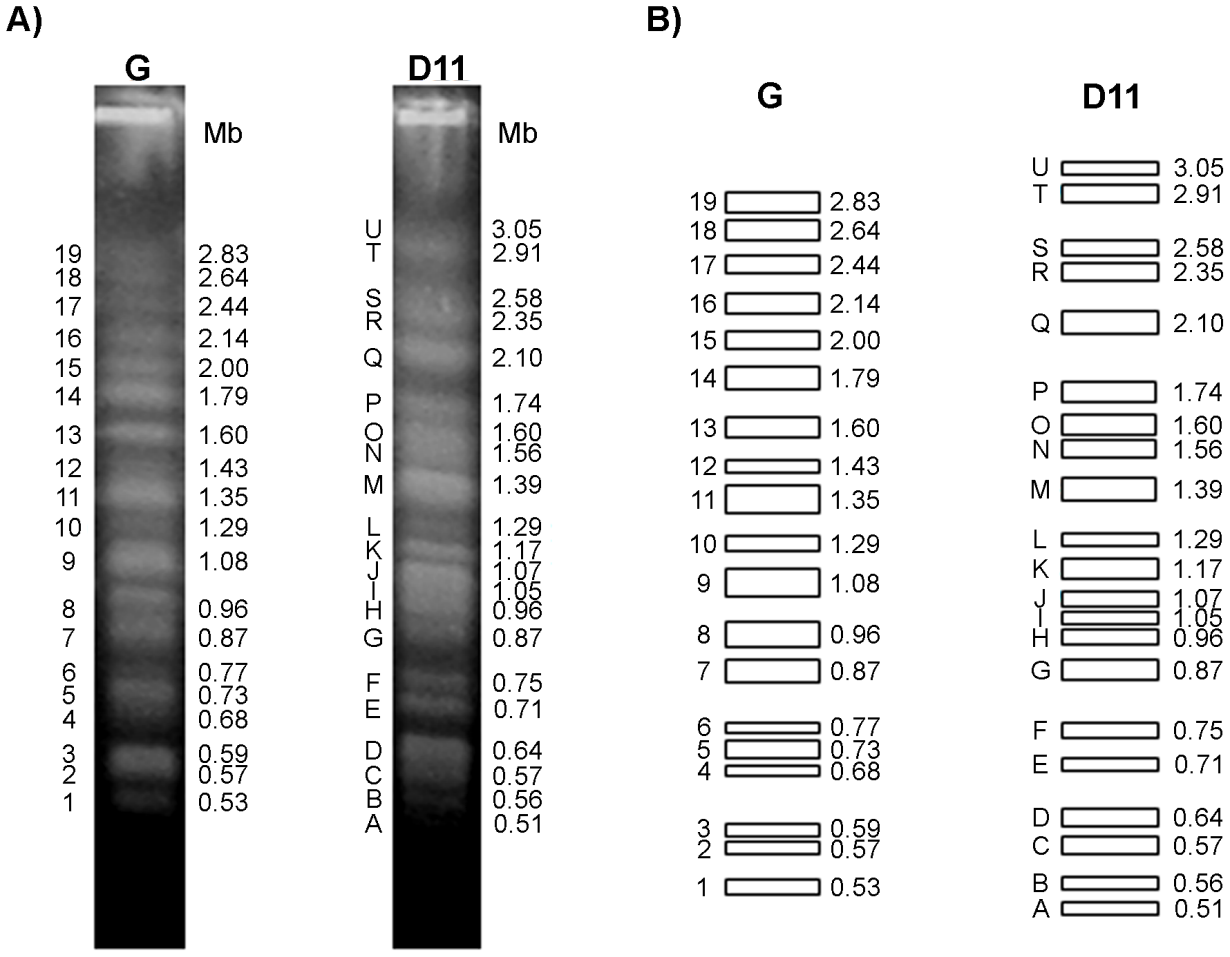
Karyotype polymorphism between the G strain and clone D11. **Panel A)** Chromosomal bands were separated by Pulsed-Field Gel Electrophoresis (PFGE) and stained with SYBR Green I. The bands from the G strain were numbered using Arabic numerals (1–19) as in a previous study (Souza et al., 2011) while capital letters (A – U) were used for clone D11, starting from the smallest band. **Panel B)** Diagrammatic representation of the molecular karyotypes of the G strain and clone D11. The rectangles represent a unique distinguishable band visualized after SYBR Green I staining. The thickness of the rectangles represents the proportional staining of each chromosomal band. The number and letter of chromosomal bands as well as their molecular weight are indicated to the left and right of each strip, respectively.

The chromosomal bands of the G strain and clone D11 were separated by PFGE and stained with EtBr (Figure 1). We refer to a DNA band visible on PFGE after staining with EtBr as a “chromosomal band”. This can contain one, two or more, not necessarily homologous, co-migrating chromosomes. EtBr staining pattern and the diagrammatic representation of chromosomal bands from the G strain and clone D11 are shown in Figures 1A and 1B, respectively. We identified 19 and 21 non-stoichiometrically staining chromosomal bands in the G strain and clone D11, respectively, by scanning pulsed-field gels stained with SYBR Green I. The molecular karyotype of the G strain is composed of 19 chromosomal bands: 11 megabase bands ranging from 2.83 to 1.08 Mb and 8 intermediate bands between 0.96 and 0.53 Mb.

The chromosome distribution of clone D11 is quite dissimilar to that of the parental G strain. We defined 21 chromosomal bands in clone D11 ranging from 3.05 to 0.51 Mb: 13 megabase bands (3.05 to 1.05 Mb) and 8 intermediate bands (0.96 to 0.51 Mb). Most of the chromosome length polymorphisms were of small amplitude, and the absence of one band in clone D11 in relation to its reference position in the G strain can be correlated to the presence of a novel band migrating above or below this position. Compared with the parental G strain, the most polymorphic bands of clone D11 were bands U (3.05 Mb) and T (2.91 Mb), which were not found in the G strain. These karyotype profiles were reproducible, were obtained repeatedly and proved to be stable in continuous culture of isolates over several years (data not shown).

**Figure 2:**
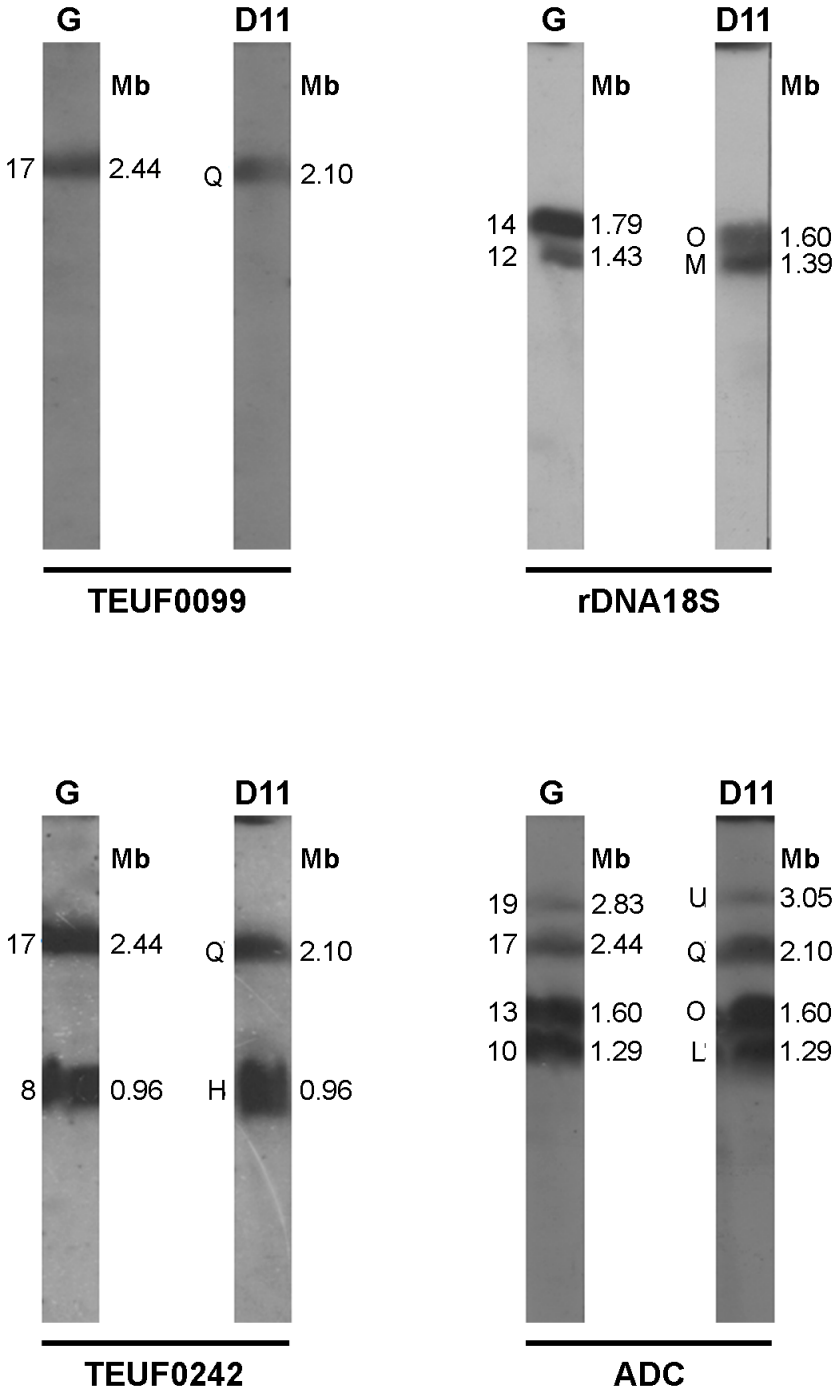
Identification of homologous chromosomal bands of similar molecular sizes in the G strain and clone D11. Hybridization profile of specific chromosomal markers hybridized to one or more bands of similar molecular size in both isolates after chromosome separation by PFGE and Southern-blot hybridization. The markers used are TEUF0099, rDNA18S, TEUF0242 and ADC. Gene identification and GenBank accession number of each marker are shown in Table 1.

**Figure 3:**
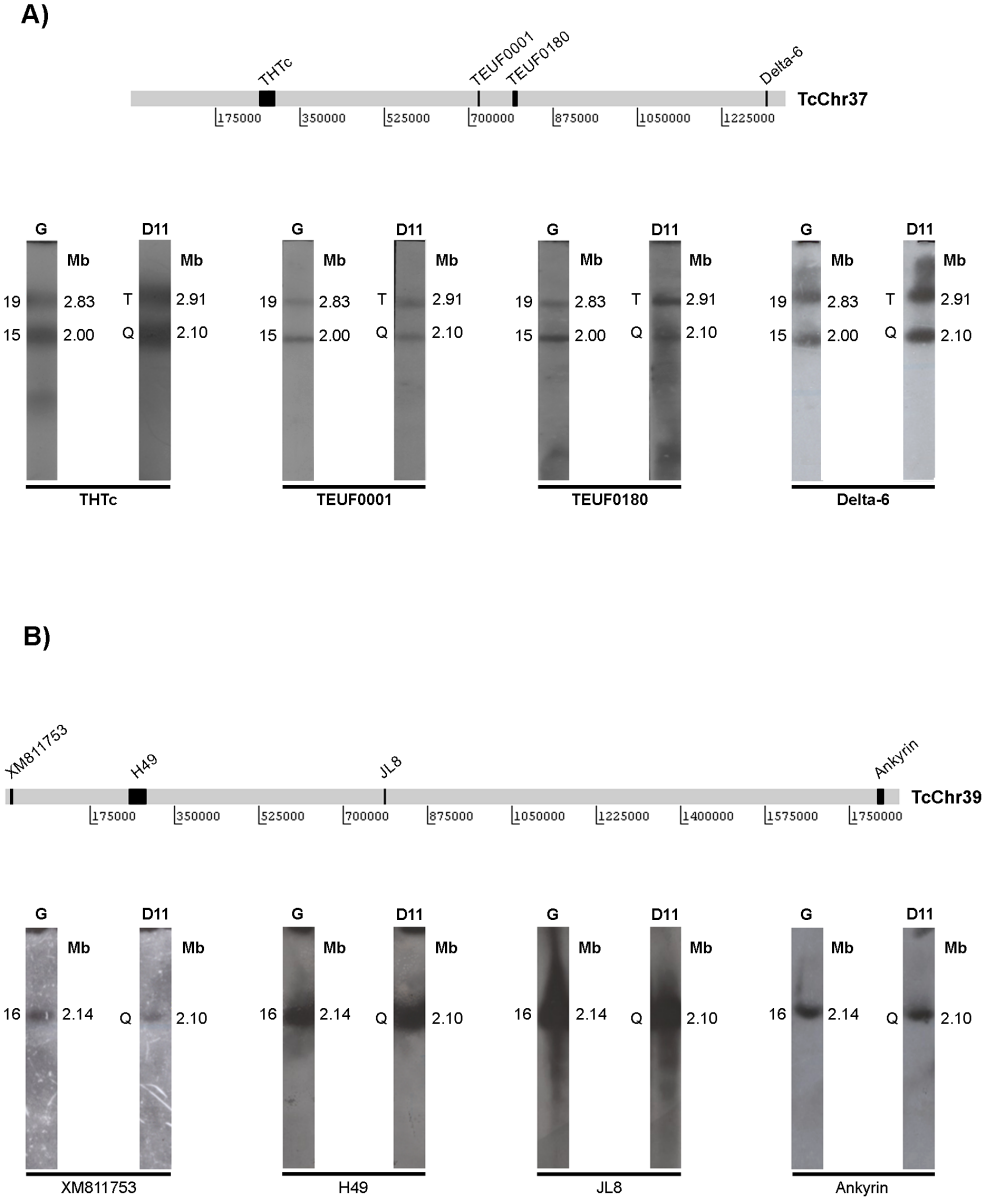
Conservation of large syntenic groups between the G strain and clone D11. Selected markers belonging to *in silico* chromosomes TcChr37 (**Panel A**) and TcChr39 (**Panel B**) previously defined in clone CL Brener were mapped on chromosomal bands of the G strain and clone D11 separated by PFGE. The diagrammatic representation above each panel indicates the position of the markers on the *in silico* chromosome. Markers from TcChr37 are THTc, TEUF0001, TEUF0180 and delta-6. Markers from TcChr39 are XM_811753, H49, JL8 and ankyrin. Gene identification and GenBank accession number of each marker are shown in Table 1.

**Figure 4:**
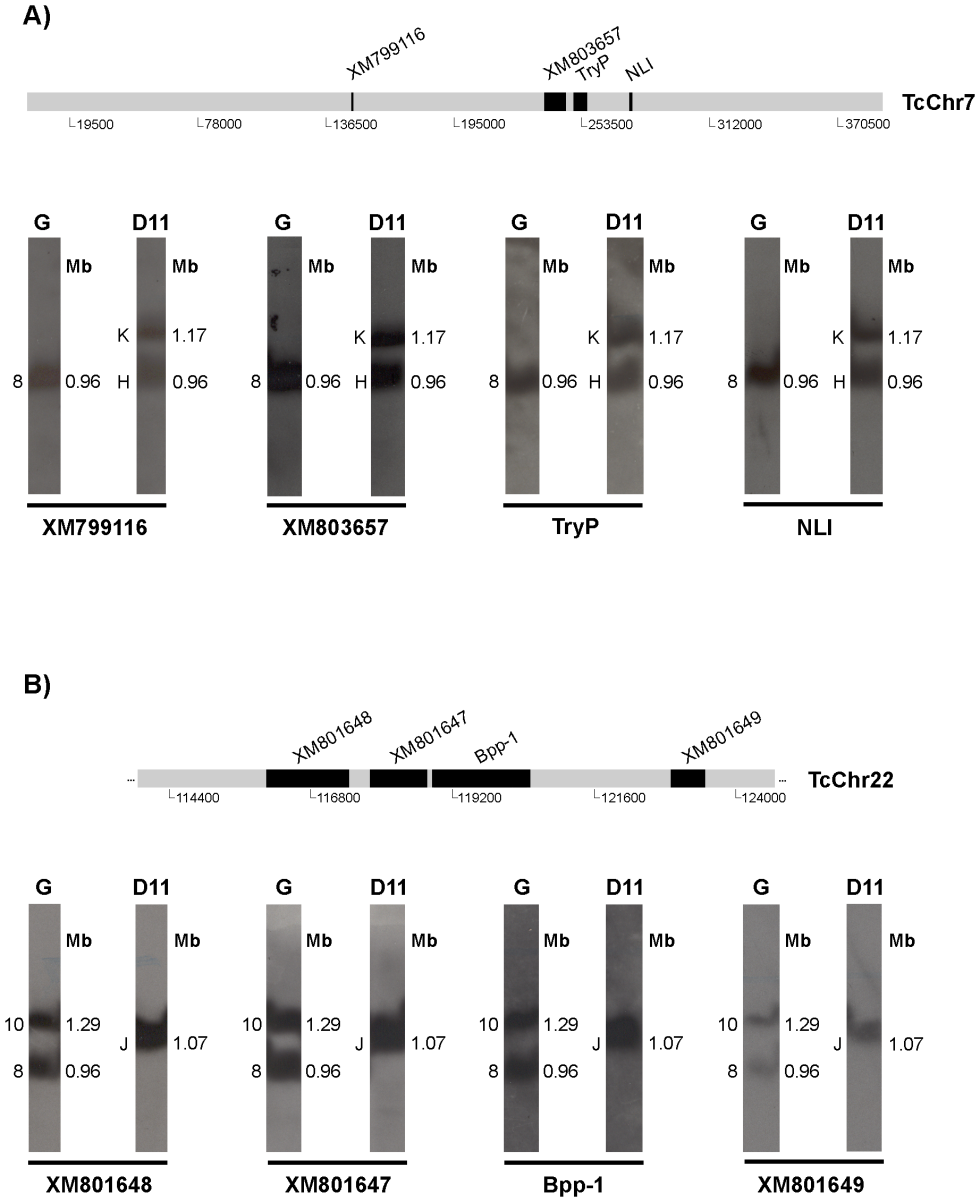
Identification of possible chromosomal rearrangements in clone D11. Mapping of markers belonging to *in silico* chromosomes TcChr7 (**Panel A**) and TcChr22 (**Panel B**). Identification of chromosomal rearrangements involving one band in the G strain and two bands in clone D11 (**Panel A**) or vice versa (**Panel B**). The positions of the markers used as radiolabeled probes are indicated in the diagrammatic representation of the *in silico* chromosomes. Markers from TcChr7 are XM_799116, XM_803657, TryP and NLI. Markers from TcChr22 are XM_801648, XM_801647, Bpp-1 and XM_801649. Gene identification and GenBank accession number of each marker are shown in Table 1.

To investigate variations in chromosome size, Southern blots were carried out and chromosomes that had been separated by PFGE were hybridized with a panel of cloned sequences (Table 1), including proteins and genes encoding ribosomal RNA, chromosome specific-markers and polymorphic repetitive sequences. The overall analysis showed distinct hybridization patterns represented by markers that hybridize to (i) one or more very similar-sized bands in both isolates (Figures 2 and 3); (ii) one band in the G strain and two bands in clone D11 or vice versa, with differences in size of up to 330 kb (Figure 4); and (iii) many bands generating a complex hybridization pattern (Figure S2).

**Table 1:** Location of molecular markers on G strain and clone D11 chromosomal bands.

Marker	Gene Identification	Accession Number	G strain#	Clone D11#
Satellite DNA	satellite DNA (195 bp)	AY520076	Several	Several
L1Tc	*T. cruzi* retrotransposon L1 Tcg62	AY112672	Several	Several
TcTREZO	*T. cruzi* clone Z25 EcoRI repeat region	AF508945	Several	Several
Transialidase	stage-specific surface glycoprotein	EF154827	Several	Several
MASP	Mucin-associated surface protein	XM_799963	Several	Several
TEUF0001	Histone H2B	AA399704	19; 15	T; Q
TEUF0099	Hypothetical protein	AA441781	17	Q
TEUF0180	85 kDa HSP	AA426667	19; 15	T; Q
TEUF0242	Unknown	AA882669	17; 8	Q; H
rDNA18S	rDNA18S	-	14; 12	O; M
ADC	Adenylate cyclase	AF031927	19; 17; 13; 10	U; Q; O;L
THTc	Hexose transporter	U05588	19; 15	T; Q
Delta-6	Delta-6 fatty acid desaturase	XM_807338	19; 15	T; Q
XM_811753	Hypothetical protein	XM_811753	16	Q
H49	Cytoskeleton-associated antigen	U16294	16	Q
JL8	Immunodominant antigen	AF147956	16	Q
Ankyrin	Ankyrin repeat protein	XM_812345	16	Q
XM_799116	Hypothetical protein	XM_799116	8	K; H
XM_803657	Hypothetical protein	XM_803657	8	K; H
TryP	Tryparedoxin peroxidase	AJ012101	8	K; H
NLI	NLI-interacting factor, putative	XM_801455	8	K; H
XM_801647	Hypothetical protein	XM_801647	10; 8	J
XM_801648	Hypothetical protein	XM_801648	10; 8	J
XM_801649	Hypothetical protein	XM_801649	10; 8	J
Bpp-1	Beta propeller protein-1	AJ577830	10; 8	J
alpha-tubulin	alpha-tubulin	L37345	15; 11	S; R
beta-tubulin	beta-tubulin	AF455117	15; 11	S; R
XM_804243	Hypothetical protein	XM_804243	15; 11	S; R
XM_812238	Endomembrane protein	XM_812238	15; 11	S; R

# Chromosomal bands separated by PFGE and stained with ethidium bromide.

Some markers hybridized to one or more bands of similar molecular size in both isolates, indicating that these chromosomes are indeed homologous (Figures 2 and 3). For instance, marker TEUF0099 hybridized to a band of 2.44 Mb in the G strain and to a band of 2.10 Mb in clone D11; marker 18S rDNA mapped with two bands of 1.43 and 1.79 Mb in the G strain and with bands 1.39 and 1.60 Mb in clone D11 (Figure 2). Chromosome size differences between the isolates were often small – up to 340 kb – suggesting small chromosome rearrangements. Although there are chromosomes of the same size in both isolates, several markers were mapped on different-sized chromosomes in both clone D11 and the G strain. For example, in clone D11 the 18S rDNA gene marker is located on the 1.60 Mb (O) and 1.39 Mb (M) chromosomes (Figure 2) while in the G strain it is located on the 1.79 Mb and 1.43 Mb chromosomes even though this strain has a 1.60 Mb chromosome.

Recently, contigs and scaffolds from clone CL Brener (reference strain of the *T. cruzi* genome project) were assembled in 41 platforms tentatively named as chromosomes (TcChr) [23]. For this reason, we would rather refer to them as *in silico* chromosomes. The linkage groups shown in Figure 3 represent two large syntenic groups conserved among isolates from different lineages of *T. cruzi* [9]. We used chromosome-specific markers that had been previously mapped on chromosomal bands XX and XVI of clone CL Brener [9]. The markers hexose transporter (THTc), TEUF0001 (histone H2B), TEUF0180 and delta-6-fatty acid desaturase were previously assigned to a single *in silico* 1.35 Mb chromosome in clone CL Brener named TcChr37. These markers hybridized with two distinct bands in the G strain (2.00 and 2.83 Mb) and clone D11 (2.10 and 2.91 Mb) (Figure 3A). The location of these markers in two chromosomal bands in both isolates (bands 2.00 and 2.83 Mb in the G strain and 2.10 and 2.91 Mb in clone D11) suggests that these bands correspond to homologous chromosomes that are shared by the G strain and clone D11 but are of different sizes.

The markers XM_811753, H49, JL8, calpain and ankyrin are located in a single *in silico* chromosome (TcChr39) approximately 1.85 Mb long in clone CL Brener. All of these markers hybridized to a chromosomal band of very similar molecular size in the G strain (2.14 Mb) and clone D11 (2.10 Mb) (Figure 3B). Hybridization of chromoblots with probes located at the opposite ends of the scaffold (XM_811753 and ankyrin) confirmed the conservation of this linkage group between the G strain and clone D11.

Using others linkage groups established in clone CL Brener, we identified chromosomal rearrangements involving one band in the G strain and two bands in clone D11 or vice versa (Figure 4). Markers XM_799116, XM_803657, tryparedoxin peroxidase (TryP) and NLI were assigned to only one *in silico* chromosome (TcChr7) approximately 0.39 Mb long in clone CL Brener. They hybridized to one chromosomal band (0.96 Mb) in the G strain and two chromosomal bands in clone D11, one of the same size (0.96 Mb) that mapped on the G strain and another 1.17 Mb long (Figure 4A). Markers XM_801648, XM_801647, beta propeller protein-1 (Bpp-1) and XM_801649 were assigned to a single *in silico* chromosome (TcChr22) approximately 0.71 Mb long in clone CL Brener. They hybridized with two bands (0.96 and 1.29 Mb) in the G strain and only one band (1.07 Mb) in clone D11. Again, chromosome size differences between the isolates were small – up to 330 kb – suggesting small chromosome rearrangements. The fact that markers from TcChr7 and TcChr22 hybridized with the same 0.96 Mb band in the G strain indicates the presence of two heterologous chromosomes of the same size in this band in the parental strain.

The distribution of three *T. cruzi* species-specific repetitive DNA sequences (the satellite DNA element and the non-LTR retrotransposons L1Tc and TcTREZO) is shown in Figure S2. Variations in karyotypes between the G strain and clone D11 were confirmed by hybridization of these probes with the chromosomal bands (Figure S2).

### Large chromosomal rearrangements

**Figure 5:**
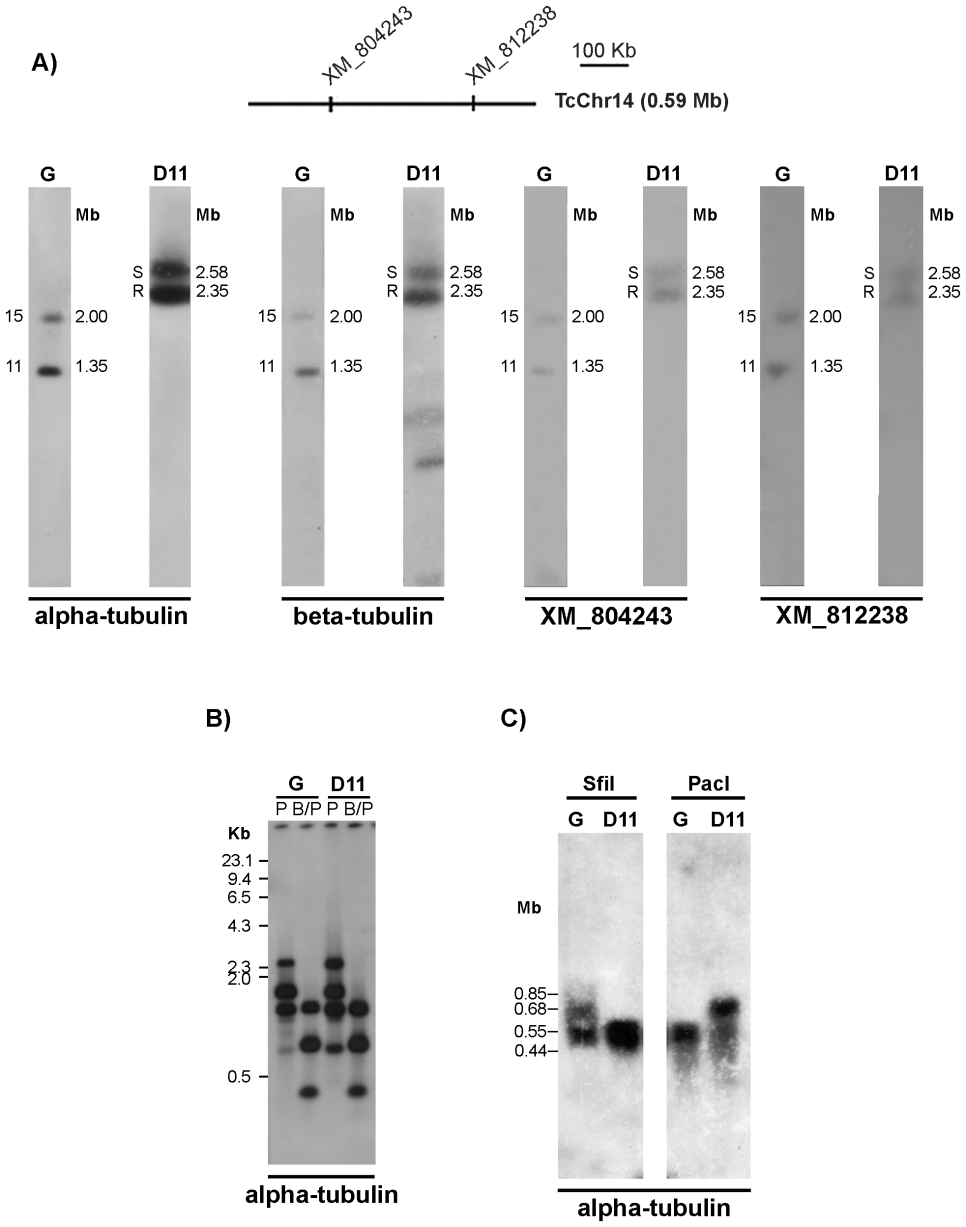
Identification of a rearrangement involving a large fragment containing the α- tubulin gene in clone D11. **Panel A**) Mapping of the α-tubulin gene on chromosomal bands of the G strain and clone D11 showing a translocation event involving large chromosomes. β-tubulin, hypothetical protein XM_804243 and endomembrane protein (XM_ 812238) were also mapped and showed the same hybridization profile. The positions of markers used as probes are indicated in the diagrammatic representation of in silico chromosomes TcChr14. **Panel B**) Restriction fragment analysis of α-tubulin gene loci was carried out by digesting genomic DNA with *Pst*I (P) or double-digesting it with *Bgl*II and *Pst*I (B/P). Phage lambda DNA digested with *Hae*III, used as a molecular weight marker, is shown on the left. **Panel C**) Restriction analysis of whole chromosomes in agarose blocks was performed using the rare-cutting enzymes *Pac*I and *Sfi*I. The molecular weights of fragments recognized by the probe are shown on the left.

The α- and β-tubulin genes were mapped on two chromosomal bands of 1.35 and 2.00 Mb in the parental G strain whereas in clone D11 they were translocated to two bands of 2.35 and 2.58 Mb (Figure 5A). Since the *T. cruzi* α- and β-tubulin genes are in physically linked as alternating tubulin repeat units ([24]; GenBank AF091836 and M97956; Bartholomeu DC, personal communication), our results suggest that the complete tubulin repeat unit was translocated to large chromosomes in clone D11. To understand this phenomenon and to investigate to what extent homologous chromosomes can be different in size, the following approaches were used: 1) hybridization of the chromoblots with probes located on the same chromosomal bands in which tubulin genes were mapped; 2) restriction analysis tubulin loci and estimation of copy number of tubulin genes in the G strain and clone D11.

We hybridized the chromoblots with probes of genes known to hybridize on the chromosomal bands of 1.35 and 2.00 Mb of G strain in which tubulin genes were mapped (Figure 5A). The markers XM_804243 and XM_812238 were located in the in silico chromosome TcChr14 at a distance of 312 kb and they hybridized with the same bands (2.35 and 2.58 Mb) recognized by tubulin probes in clone D11, confirming the occurrence of translocation of large chromosome fragments.

Next we performed restriction analysis of the tubulin loci in both isolates. Total DNA of the G strain and clone D11 was digested with restriction enzymes flanking the α-tubulin loci and hybridized with the α-tubulin gene (Figure 5B). The hybridization profiles of the parental strain and clone D11 were almost identical, suggesting that it is unlikely that amplification and/or deletion of α-tubulin tandem repeats account for the chromosome size changes. This hypothesis was further supported by the finding that the copy number of α-tubulin genes was very similar between the G strain and clone D11 (data not shown). Next, we used rare-cutting restriction enzymes to show differences between G strain and clone D11 chromosomes. Whole chromosomes enclosed in agarose blocks were digested with *Pac*I and *Sfi*I (which do not cut within the tandem repeats of the tubulin gene cluster), run on an agarose gel and hybridized with the α-tubulin probe (Figure 5C). EtBr staining of the gel indicated that the majority of the digested DNA ranged in size from 10 to 1100 kb and produced a different pattern with each enzyme (data not shown). The α-tubulin probe hybridized with two *Sfi*I restriction fragments of around 0.55 and 0.68 Mb in the G strain, and a large broad band ranging from 0.44 to 0.60 Mb in clone D11. The fragments of between 0.44 and 0.60 Mb detected in clone D11 could easily be visualized in a short-exposure autoradiograph (Figure 5C). In three independent experiments the 0.68 Mb *Sfi*I fragment faintly hybridized with the probe, suggesting the presence of a few copies of the α-tubulin gene in this fragment. The α-tubulin gene was mapped on one strongly hybridizing *Pac*I restriction fragment of about 0.55 Mb in the G strain but on a 0.68 Mb fragment in clone D11. These results suggest that chromosomal rearrangements occurred with chromosomes carrying the α-tubulin loci. Taken together these results suggest the occurrence of translocation of large chromosome fragments carrying the tubulin genes rather intrachromosomal amplification.

### Telomere length differences between the G strain and clone D11

**Figure 6:**
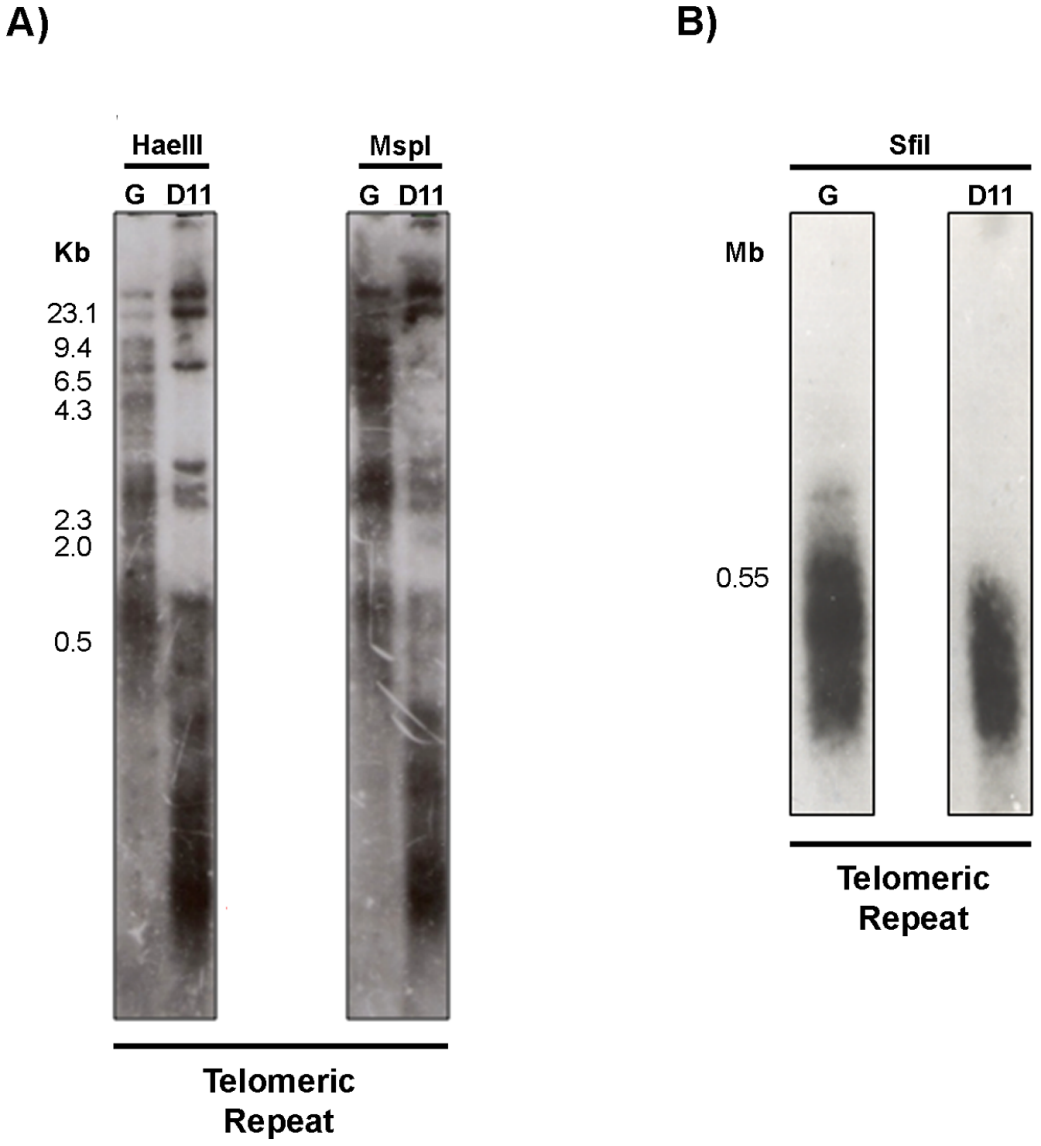
Telomere length polymorphism of the G strain and clone D11. **Panel A**) Southern-blot hybridization of restriction fragments generated by *Hae*III and *Msp*I probed with the telomeric repeat (TTAGGG). *Hae*III-digested phage lambda DNA (used as a molecular weight marker) is shown on the left. **Panel B**) Analysis of the subtelomeric length of the G strain and clone D11 chromosomes was performed by Southern-blot hybridization of *Sfi*I restriction fragments with the telomeric repeat. The size of the larger subtelomeric fragment of clone D11 is shown on the left.

To examine telomere length polymorphism in the G strain and clone D11, Southern blot hybridization was performed using frequently cutting restriction enzymes (*Hae*III and *Msp*I) whose sites are found within the telomeric junction sequence, a *T. cruzi* telomere signature. Telomeric hexameric repeats (TTAGGG) were used as a probe. The hybridization profile of the G strain was quite different from that of clone D11. The telomeric restriction fragments of the parental strain show a broad spectrum of lengths ranging from 0.5 kb to over 23 kb. With *Hae*III, bands that varied from 0.5 to approximately 3 kb were observed, representing a significant fraction of the G strain telomeres (Figure 6A). For clone D11, two distinct classes of telomeric restriction fragment were identified: one composed of fragments larger than 6.5 kb and another composed of fragments smaller than 4.3 kb. The hexameric probe hybridized only to three *Hae*III and two *Msp*I restriction fragments of over 6.5 kb, showing that either clone D11 has few large telomeres or these fragments represent internal telomeric sequences. However, only three bands ranging from 2.3 to 4.3 kb were observed for the second class of telomeric restriction fragment. It is noteworthy that in clone D11, fragments<0.5 kb hybridize strongly with the telomeric probe (Figure 6A) suggesting that most of clone D11 telomeres are less than approximately 500 bp long. The telomere shortening in clone D11 may represent a trace of earlier recombinogenic activity.

Next, the *Sfi*I rare-cutting restriction enzyme was employed to estimate the length of subtelomeric regions in both isolates. An extended smear was generated by hybridization of the *Sfi*I restriction fragments with the hexameric repeats (Figure 6B).The G strain exhibited a broader range signal than clone D11, suggesting that the polymorphism extends to subtelomeric regions. These results indicate that the chromosome length polymorphism observed between the G strain and clone D11 may be in part due to telomere length polymorphism.

### Genotyping analysis of the G strain and clone D11

The D11 variant karyotype may be associated with chromosome rearrangements during the cloning process. Alternatively, this clone may have been isolated from a pre-existing mixed population. In an attempt to answer this question, we analyzed the genetic polymorphism of clone D11 and the G strain using sequences from 10 microsatellite loci. The chromatograms were edited using AlleleLocator software, which allows the amplified fragments to be detected in the form of peaks. For a diploid organism such as *T. cruzi*, the presence of one- or two-peak patterns indicating homozygosity or heterozygosity, respectively, is expected. In this case, the *T. cruzi* strain is classified as a monoclonal strain, composed by a single population. The detection of a pattern with more than two peaks could indicate a multiclonal or aneuploid *T. cruzi* strain.

Table 2 shows the allele sizes for ten microsatellite loci in the G strain and clone D11. Three loci (SCLE11, TcTAC15 and TcAAAT6) had the same alleles in the G strain and in clone D11, and two loci (SCLE10 and ATT14) showed one allele common to the G strain and clone D11. However, the allele sizes for the other five loci (MCLE01, MCLG10, MCLF10, TcTAT20 and TcAAT8) were completely different in the G strain and clone D11. These findings suggest that the G strain has a monoclonal population structure and that its genome differs from that of the clone D11. We also performed a PCR to amplify the D7 divergent region from the 24SαrDNA gene. Both the strain and clone showed the ∼110 bp amplicon characteristic of *T. cruzi* I.

**Table 2:** Allele sizes (bp) for each microsatellite locus amplified for the G strain and clone D11.

	Allele sizes (bp)	
Locus	G strain	clone D11
SCLE11^a^	146/146	146/146
TcTAC15^a^	96/96	96/96
TcAAAT^a^	239/239	239/239
SCLE10^b^	251/251	251/259
TcAAT14^b^	250/250	250/256
MCL01^c^	136/136	128/141
MCLG10^c^	153/153	155/155
MCLF10^c^	186/186	184/190
TcTAT^c^	186/186	190/193
TcAAT^c^	229/229	241/253

a Microsatellite loci with the same alleles in the G strain and D11 clone.

b Microsatellite loci with a common allele in the G strain and D11 clone.

c Microsatellite loci with different alleles in the G strain and D11 clone.

To further investigate the monoclonality of the G strain indicated by the results of microsatellite analysis, we decided to clone this strain using another cell-cloning procedure. Serial dilutions of G-strain epimastigotes were seeded onto 96-well plates containing LIT medium supplemented with 10% human blood to obtain 1 parasite for each two wells. After 20–30 days, epimastigotes were detected in some wells, collected and seeded in LIT liquid medium for expansion. Six clones were isolated and analyzed by PFGE and hybridization, which showed that all of them were identical to the parental G strain (data not shown).

## Discussion

### Overall comparison of the genome structures of the G strain and clone D11

Although the parasite's molecular karyotype seems to be relatively stable while it is maintained in the laboratory [25], chromosomal polymorphisms can be detected and probably emerge as a result of stressful conditions. This is of particular significance because *T. cruzi* does not undergo meiosis and the parasite generally reproduces asexually. There is considerable evidence to suggest a clonal population structure [17], [26], [27], as a result of which genetic variability could only be generated during the diploid cell cycle without the involvement of gametes.

It has been shown that total DNA content can vary among *T. cruzi* isolates [8], [9], [28]–[30], suggesting that the *T. cruzi* genome is plastic. However, we estimated total DNA content of the G strain and clone D11 by the dsDNA quantitation method, and hierarchical ANOVA failed to reveal any statistically significant difference (P<0.05), suggesting that the chromosomal polymorphism is not due to DNA content but to genomic organization.

A possible explanation for this phenomenon is the central role played by repetitive sequences in the genome shape as demonstrated in *S. cerevisiae* [31], in which most of the detectable chromosomal breaks were repaired by homologous recombination with the particular involvement of Ty retrotransposon sequences, leading, in most cases, to chromosomal aberration. The *T. cruzi* genome is very rich in repetitive sequences, such as satellite DNA, retrotransposons and repeated gene families, which together comprise approximately 50% of the CL Brener genome [32].

Although the copy number of repetitive sequences differs between clone D11 and the G strain, the higher copy number found for the repetitive sequences tested here is not enough to explain the reduction in genome size observed in clone D11 (about 10 Mb). Other noncoding repetitive DNA elements, such as micro- and minisatellites and large gene families of surface proteins may account for some of the difference in genome sizes. Using comparative genomic hybridization (CGH), Minning et al. (2011) identified several CNVs (copy number variation) and aneuploidy in twelve different isolates of *T. cruzi* [33]. They observed that these polymorphisms were more frequent in repetitive-rich regions and multigene families.

### Comparison of karyotype and chromosome structure of the G strain and clone D11

Estimated chromosome size differences between clone D11 and its parental strain were relatively small (around 340 kb), suggesting small chromosome rearrangements. The fact that the variant chromosomes have a homologue of similar size in the parental strain suggests that they might be the result of DNA amplification/deletion events rather than interchromosomal exchange. Regardless of chromosomal polymorphism, we showed that large syntenic groups are conserved between the G strain and clone D11. Two large syntenic groups of 1.35 Mb (*in silico* chromosome TcChr37) and 1.85 Mb (*in silico* chromosome TcChr39) were mapped on chromosomal bands with similar sizes in clone D11 and the G strain (Figure 3), suggesting the maintenance of gene order and a striking conservation of chromosome structure in clone D11. The location of TcChr37 markers in two chromosomal bands (2.00 and 2.83 Mb in the G strain; 2.10 and 2.91 Mb in clone D11) could be explained by the existence of two different-sized homologous chromosomes or the occurrence of a large duplication event comprising the 1.36 Mb regions of two non-homologous chromosomes.

**Figure 7:**
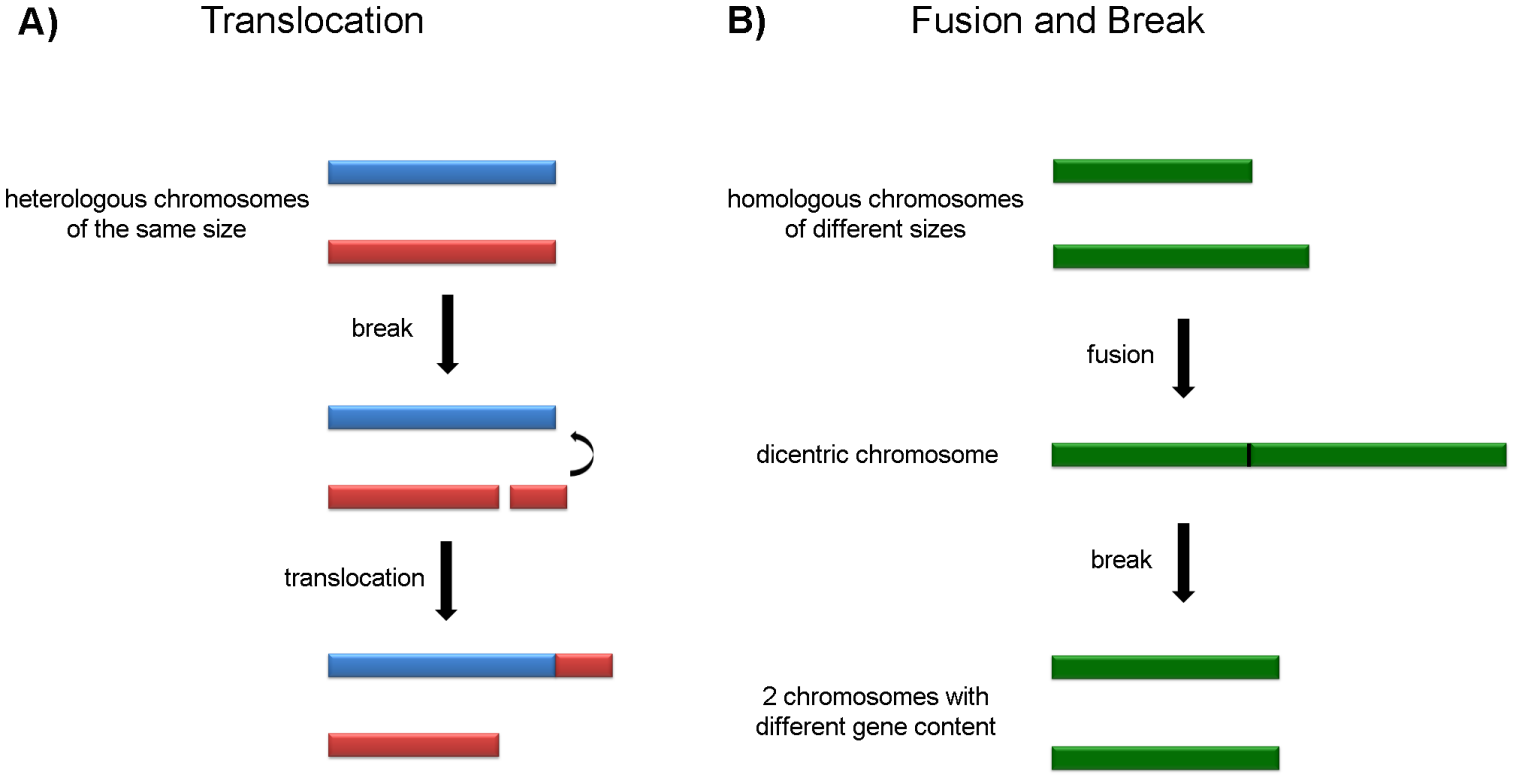
Possible mechanisms of genetic recombination that could give rise to chromosomal polymorphism in *T.* *cruzi*. **Panel A**) Translocation mechanism: a DNA fragment (210 kb) from a heterologous chromosome (red) is translocated to another chromosome (blue) by homologous recombination, generating “homologous” chromosomes of different sizes. **Panel B**) Fusion and breakage mechanism: two homologous chromosomes of different sizes are fused, forming a dicentric chromosome which is then broken, generating two chromosomes of similar sizes but with different gene content.

The other syntenic group (TcChr7) was assigned to one single chromosomal band in the G strain and two similar-sized bands in clone D11 that may correspond to size-polymorphic homologous chromosomes. A number of different mechanisms may be responsible for the chromosome size polymorphism. The hybridization of the same markers in two distinct bands in clone D11 leads us to speculate that repetitive sequences may have been amplified or that a chromosomal fragment of approximately 210 kb may have been translocated in one of the homologues, possibly by a similar mechanism to that suggested in Figure 7.

To explain the hybridization of chromosome markers of TcChr22 in two chromosomal bands in the G strain (0.96 and 1.29 Mb) and only one in clone D11 (1.07 Mb), we hypothesized that chromosomes of the G strain fused to give rise to a dicentric chromosome and that this was followed by breakage to generate two chromosomes of similar size (approximately 1.07 Mb) in clone D11 (Figure 7). The "new" chromosomes may have partially altered gene content. In the case of the tubulin genes, the evidence suggests a model based on interchromosomal exchange of large segments of DNA.

Another source of chromosomal polymorphism resides at the chromosome termini. Telomeric and subtelomeric regions are hotspots for recombination events in several unicellular microorganisms [34]–[37]. Telomere length variation has been described in different *T. cruzi* strains. Strains Y, Berenice and F possess telomeres ranging in size from 0.5 to 1.5 kb, while clones CL Brener and Dm28c have significantly larger telomeres (1 to 10 kb) [38]. We found that clone D11 has very small telomeres compared with the G strain. This phenomenon could be the result of many cell cycle replications occurring in the absence of telomerase activity or defects in components of the telomeric chromatin, which in mammals and yeast culminate in telomere shortening or fusion, with the formation of dicentric chromosomes [39], [40]. In another round of cell replication, telomere fusions may trigger chromosomal breakage and aneuploidy [39], [40]. Another possibility would be the occurrence of chromosomal breaks, possibly in a genomic region rich in repetitive sequences such as the transialidase superfamily, followed by telomerase-mediated healing, which may add a few units of telomeric repeats and generate new short telomeres, as in the model proposed by [41].

### Could the D11 variant karyotype have arisen during the cloning process?

The question remains whether the parental G strain is a heterogeneous *T. cruzi* population and karyotypic variants preexisted in the parental population or chromosomal rearrangements occurred during the cloning process. Allele analysis using sequences from ten microsatellite loci indicated that the G strain has a monoclonal structure. However, we cannot rule out the hypothesis of the existence of a multiclonal population structure formed by underrepresented individuals that are genetically different from the original strain. If this is the case, clone D11 could be a less representative subpopulation of the G strain that microsatellite PCR and PFGE analysis would not be sensitive enough to detect.

Chromosomal rearrangements have been shown to occur during the cloning process in several protozoan parasites such as *Giardia lamblia* [42], *Leishmania* ssp [43]–[46] and *T. cruzi* [10]. The chromosomal heterogeneity observed in clones originated from a single strain could be explained by the occurrence of chromosomal DNA rearrangements and/or the presence of a multiclonal strain, with slight differences between the clones, but with a predominant population, as suggested by [43], [45], [46]. In this case, the strain displays a mosaic structure with different cells possessing homologous chromosomes of different sizes due to frequent DNA amplification/deletion events [43], [45], [46].

In *T. cruzi*, McDaniel and Dvorak (1993) [10] reported that clones with the same isoenzymic and schizodeme profiles differ in their DNA content. Campos and Andrade (1996) [47] showed that clones and subclones displayed the same isoenzymic patterns and biological behavior similar to the parental strain, with minor variability in the parasitemic profiles. These results could be explained by the mosaic strain concept suggested by [43], [46].

The evidence reported here does not allow us to define whether clone D11 stems from a homogeneous monoclonal strain and was induced by the stress of the cloning procedure or it emerged from a multiclonal, mosaic strain. In the latter case, the variant represented by clone D11 would be better adapted to survive the stress induced by cloning, which includes intracellular development in the mammalian cell. It is interesting to note that cloning of the G strain by a less stressful procedure, i.e., serial dilution and plating in soft agar, resulted in clones that were identical to the parental strain. The results presented in this manuscript highlight the complexity of the genetic structure of *T. cruzi* populations and the difficulties involved in carrying out a more in-depth analysis of the mechanisms underlying chromosome rearrangements in this parasite. Comparative analysis of the G strain and clone D11 will be carried out in our laboratory using comparative genomic hybridization (CGH) to elucidate these mechanisms.

## Supporting Information

Figure S1 **Flow cytometry analysis showing DNA synchronization after hydroxyurea (HU) treatment.** Panels **A** and B present, respectively, flow cytometry analysis of G strain and clone D11 epimastigotes stained with propidium iodide. Histograms of non-treated cells are presented on the left and those treated with 20 mM HU are presented on the right. The number above the first peak corresponds to the percentage of cells in G1 phase and that above the second peak to the S/G2 phase.
(TIF)

Figure S2 **Distribution of repetitive elements on chromosomal bands of the G strain and clone D11.** Chromosomal bands were separated by PFGE and hybridized with the satellite DNA, non-LTR retrotransposon L1Tc, TcTREZO, transialidase and mucin-associated surface protein (MASP), generating a complex hybridization pattern. Gene identification and GenBank accession number of each marker are shown in Table 1.
(TIF)
