# Supplementary figures and images for: Interclonal Variations in the Molecular Karyotype of Trypanosoma cruzi: Chromosome Rearrangements in a Single Cell-Derived Clone of the G Strain
Source: PLoS One. 2013 May 7;8(5):e63738. doi: 10.1371/journal.pone.0063738 (PMC3646811; doi:10.1371/journal.pone.0063738)

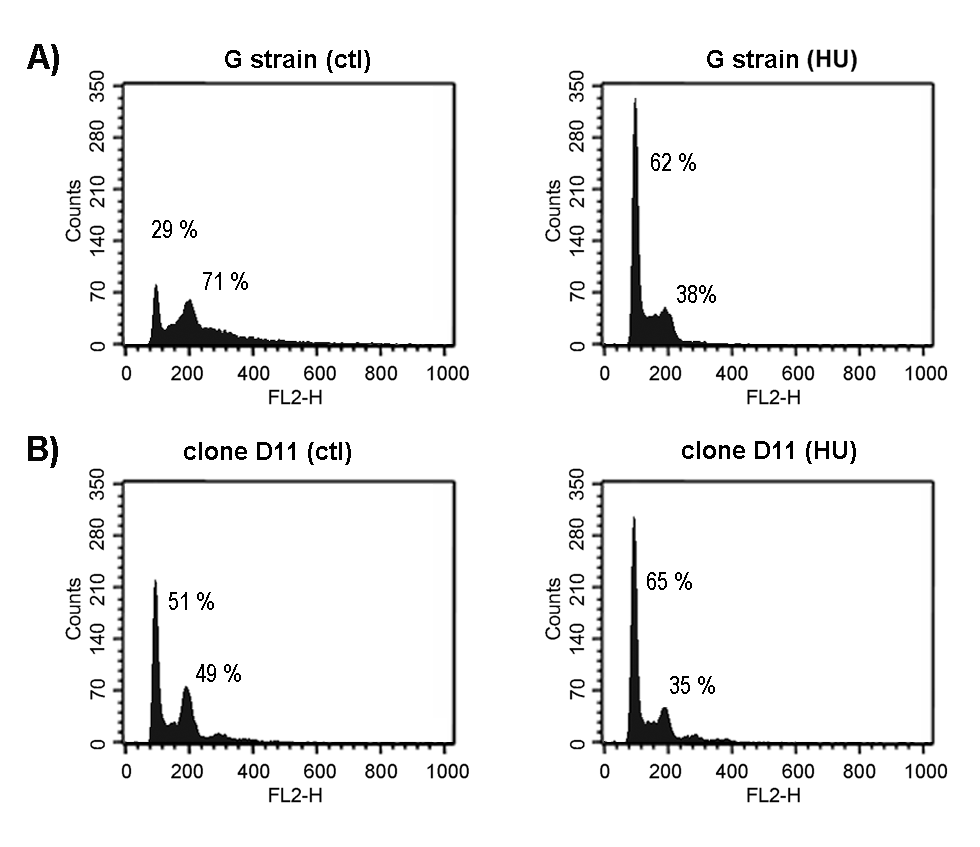

Supplement: Figure S1 — Flow cytometry analysis showing DNA synchronization after hydroxyurea (HU) treatment. Panels A and B present, respectively, flow cytometry analysis of G strain and clone D11 epimastigotes stained with propidium iodide. Histograms of non-treated cells are presented on the left and those treated with 20 mM HU are presented on the right. The number above the first peak corresponds to the percentage of cells in G1 phase and that above the second peak to the S/G2 phase. (TIF) [file pone.0063738.s001.tif]

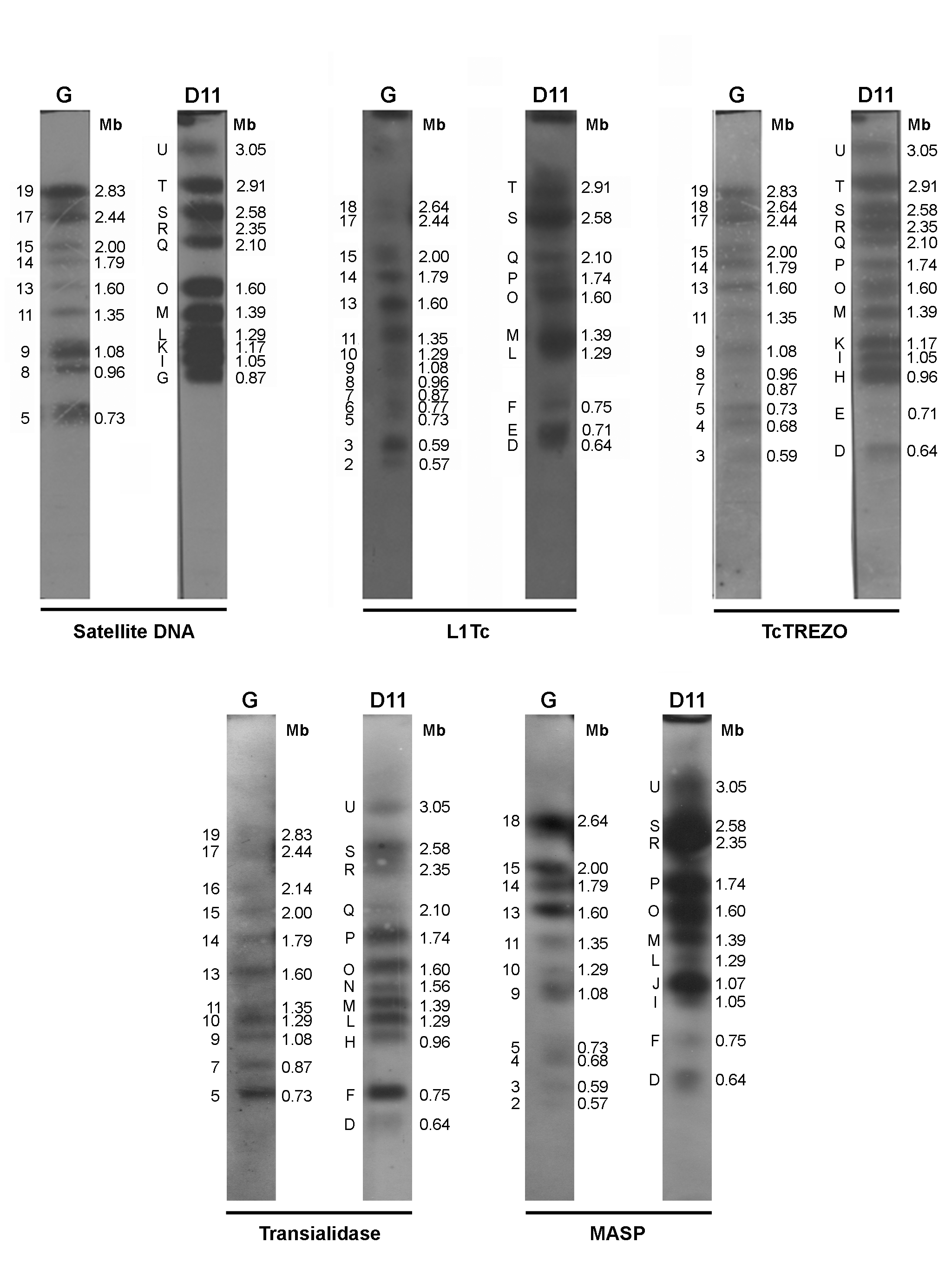

Supplement: Figure S2 — Distribution of repetitive elements on chromosomal bands of the G strain and clone D11. Chromosomal bands were separated by PFGE and hybridized with the satellite DNA, non-LTR retrotransposon L1Tc, TcTREZO, transialidase and mucin-associated surface protein (MASP), generating a complex hybridization pattern. Gene identification and GenBank accession number of each marker are shown in Table 1. (TIF) [file pone.0063738.s002.tif]
